# Silicon Alleviates Drought Stress and Enhances Rice Seedling Establishment Under Simulated Dry Direct Seeding via Regulation of ABA and JA Signaling

**DOI:** 10.3390/plants15121813

**Published:** 2026-06-12

**Authors:** Yanyan Sun, Yinuo Ma, Shijie Wei, Lanfang Zhang, Kaixiang Tao, Zishu Xu, Rongjun Zhang, Xinyu Chen, Long Li, Yuanyuan Song, Long Lu, Rensen Zeng

**Affiliations:** 1 State Key Laboratory of Agricultural and Forestry Biosecurity, Key Laboratory of Ministry of Education for Genetics, Breeding and Multiple Utilization of Crops, College of Agriculture, Fujian Agriculture and Forestry University, Fuzhou 350002, China; yanyansun565340120@163.com (Y.S.); mayinuo913@163.com (Y.M.); 15778312359@163.com (S.W.); 15386743512@163.com (L.Z.); tkaixiang@163.com (K.T.); 18846192767@163.com (R.Z.); chenxinyu_fafu@163.com (X.C.); 18625356805@163.com (L.L.); yyuansong@fafu.edu.cn (Y.S.); 2 Key Laboratory of Biological Breeding for Fujian and Taiwan Crops, Ministry of Agriculture and Rural Affairs, Key Laboratory of Crop Biotechnology of Fujian Higher Education Institutes, Fujian Agriculture and Forestry University, Fuzhou 350002, China

**Keywords:** dry direct seeding, silicon, drought, abscisic acid, jasmonic acid, root architecture

## Abstract

Dry direct seeding (DDS) is a water-saving and high-efficiency rice cultivation system. However, drought stress during DDS severely constrains seedling establishment. This study used the conventional rice variety Zhonghua 11 (ZH11) and the drought-tolerant hybrid Hanyou 73 to investigate the effects of exogenous silicon (Si) on seed germination and seedling growth under drought stress, and to explore the underlying mechanisms of Si-enhanced drought tolerance. Drought stress was imposed using PEG-6000 simulation and pot experiments with different soil relative water contents (60%, 45%, 25%, and 10%). Si treatment significantly alleviated simulated drought inhibition of seed germination, increasing germination percentage and index, improving seedling growth in both varieties. Under simulated DDS conditions, Si significantly improved plant height, biomass, and root development, while maintaining higher net photosynthetic rate, stomatal conductance, intercellular CO_2_ concentration, transpiration rate, and chlorophyll content. Meanwhile, Si reduced oxidative damage by promoting proline accumulation, enhancing peroxidase (POD) and catalase (CAT) activities in both leaves and roots, reducing malondialdehyde (MDA) accumulation, and upregulating the expression of key drought-responsive genes (*SNAC1*, *DREB1A*, *SKIPa*, *P5CS2*). Furthermore, Si upregulated the expression of genes involved in abscisic acid (ABA) (*ABA1*, *ABA2*, *MHZ5*, *ABI3*) and jasmonic acid (JA) (*AOS2*, *AOS3*, *JAR1*, *JAR2*, *MYC2*, *COI1a*) biosynthesis and signaling. Compared with the wild-type, the ABA signaling mutant *abi3* and the JA signaling mutant *myc2* exhibited significantly attenuated improvement of plant growth by Si treatment. Collectively, Si enhances antioxidant capacity and osmotic adjustment, maintains photosynthetic function, and is associated with the activation of ABA and JA signaling pathways, which together alleviate the inhibition of rice seedling establishment under DDS-associated drought stress. Our findings provide a theoretical basis for the application of Si fertilizer in DDS rice production.

## 1. Introduction

The dry direct seeding (DDS) cultivation system is being rapidly adopted in rice-growing regions of southern China due to its significant advantages in water savings, labor efficiency, high productivity, and compatibility with mechanization, making it an important strategy for maintaining stable rice cropping areas [1,2]. However, DDS rice often experiences varying degrees of drought stress during the period from sowing to emergence. Drought at the seedling stage inhibits seed germination, reduces emergence rate, and suppresses seedling growth, which in turn affects seedling density and uniformity, severely compromising the high and stable yield of DDS rice [3]. Therefore, deciphering the mechanisms underlying the response of DDS rice to drought stress at the seedling stage is of great practical importance for the genetic improvement of drought-tolerant varieties.

Silicon (Si) is the second most abundant element in the Earth’s crust, and its accumulation in rice and other graminaceous crops can reach up to 10% of dry weight [4]. Numerous studies have shown that exogenous Si application significantly enhances plant tolerance to various abiotic stresses [5,6]. Under drought stress, Si application alleviates the inhibition of rice growth through multiple mechanisms, including maintaining leaf relative water content, increasing photosynthetic rate, promoting the accumulation of osmotic regulatory substances (such as proline), enhancing the activities of antioxidant enzymes (such as peroxidase (POD) and catalase (CAT)), and reducing the accumulation of malondialdehyde (MDA) [7,8,9]. In addition, Si can enhance root water uptake from deeper soil layers by modulating root architecture [8]. Nevertheless, most existing studies have focused on conventionally irrigated or transplanted rice systems, and the mechanisms of Si action in DDS rice under different soil water contents remain poorly understood [9].

The phytohormones abscisic acid (ABA) and jasmonic acid (JA) both play important roles in regulating drought stress responses [10,11]. ABA enhances plant drought tolerance by inducing stomatal closure, reducing water transpiration, and activating downstream stress-responsive gene expression [10]. JA has also been shown to participate in drought stress regulation, improving plant drought tolerance by promoting root development and enhancing antioxidant activity [11,12]. Several studies have shown that Si can enhance drought tolerance in rice through the ABA signaling pathway [13] and that Si accumulation is also regulated by the JA signaling pathway [14]. However, whether Si enhances drought tolerance through the ABA and JA pathways under simulated DDS conditions, and the respective contributions of these two pathways to Si-mediated drought tolerance, have not been systematically validated using genetic approaches.

Despite extensive reports on the positive effects of silicon on drought tolerance in rice, several key gaps remain, including a poor understanding of the mechanisms of Si action under DDS conditions with varying soil water contents, a lack of systematic genetic validation of whether Si enhances drought tolerance through both ABA and JA pathways, and unclear respective contributions of these two pathways to Si-mediated drought tolerance. To address these gaps, this study systematically investigated the effects of Si application on seed germination, seedling growth, photosynthetic characteristics, antioxidant defense, and osmotic regulation under simulated DDS conditions, analyzed the regulatory effects of Si on the expression of genes involved in ABA and JA synthesis and signal transduction, and used the ABA signaling mutant *abi3* and the JA signaling mutant *myc2* to validate the functions of these two signaling pathways in Si-mediated drought tolerance. This study aims to elucidate the physiological and molecular mechanisms by which Si application regulates drought tolerance in rice, clarify the roles of the ABA and JA signaling pathways in this process, and provide a theoretical basis for the rational application of Si fertilizer in DDS cultivation systems.

## 2. Results

### 2.1. Silicon Alleviates the Inhibition of Seed Germination and Early Seedling Growth Under Simulated Drought Stress in Rice

**Figure 1:**
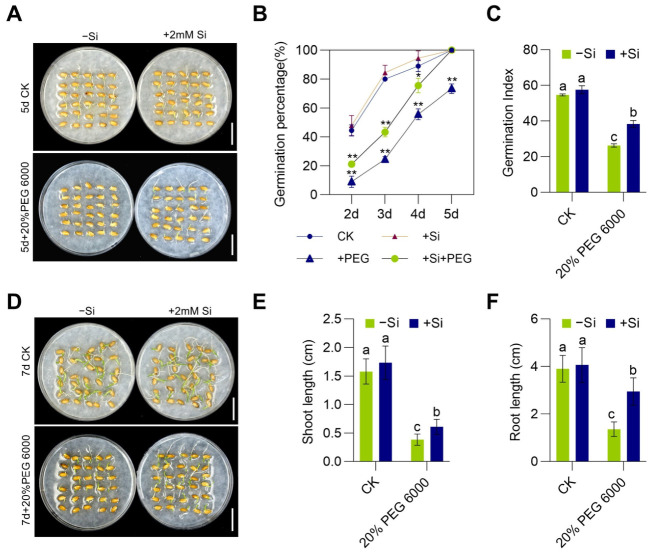
Si alleviates the inhibition of ZH11 rice seed germination and early seedling growth under simulated drought stress. (**A**) Phenotypic images of rice seeds germinated for 5 days under normal conditions (CK) or 20% PEG 6000 treatment, with or without 2 mM Si addition. Scale bars, 2 cm. (**B**) Germination percentage of rice seeds at 2–5 days after treatment under different conditions (CK, +Si, +PEG, +Si +PEG). (**C**) Germination index of rice seeds after 5 days of treatment under CK or 20% PEG 6000, with or without Si addition. (**D**) Phenotypic images of rice seedlings grown for 7 days under CK or 20% PEG 6000 treatment, with or without 2 mM Si addition. Scale bars, 2 cm. (**E**,**F**) Shoot length (**E**) and root length (**F**) of rice seedlings after 7 days of treatment under the indicated conditions. (**B**,**C**) Data are presented as mean ± SD (n = 3). (**E**,**F**) Data are presented as mean ± SD (n = 15). Asterisks indicate significant differences between CK and treatment groups under the same treatment (* *p* < 0.05, ** *p* < 0.01, Student’s *t*-test). Different lowercase letters indicate significant differences among groups (*p* < 0.05, one-way ANOVA followed by Tukey’s test).

**Figure 2:**
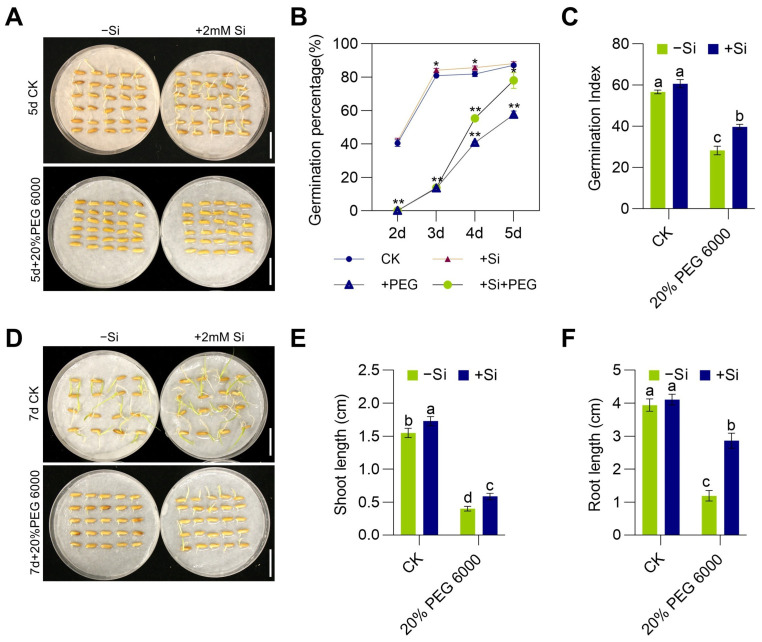
Si alleviates the inhibition of drought-tolerant hybrid rice variety Hanyou 73 rice seed germination and early seedling growth under simulated drought stress. (**A**) Phenotypic images of rice seeds germinated for 5 days under normal conditions (CK) or 20% PEG 6000 treatment, with or without 2 mM Si addition. Scale bars, 2 cm. (**B**) Germination percentage of rice seeds at 2–5 days after treatment under different conditions (CK, +Si, +PEG, +Si +PEG). (**C**) Germination index of rice seeds after 5 days of treatment under CK or 20% PEG 6000, with or without Si addition. (**D**) Phenotypic images of rice seedlings grown for 7 days under CK or 20% PEG 6000 treatment, with or without 2 mM Si addition. Scale bars, 2 cm. (**E**,**F**) Shoot length (**E**) and root length (**F**) of rice seedlings after 7 days of treatment under the indicated conditions. (**B**,**C**) Data are presented as mean ± SD (n = 3). (**E**,**F**) Data are presented as mean ± SD (n = 15). Asterisks indicate significant differences between CK and treatment groups under the same treatment (* *p* < 0.05, ** *p* < 0.01, Student’s *t*-test). Different lowercase letters indicate significant differences among groups (*p* < 0.05, one-way ANOVA followed by Tukey’s test).

Under 20% PEG treatment, after 5 days of treatment, the germination percentage of ZH11 decreased from 100% (CK) to 73.3%. Si application significantly alleviated this inhibition, increasing the germination percentage to 100% (Figure 1A,B). Similarly, the germination index was reduced by 28.36% under drought, but Si treatment restored it by 12.02% (Figure 1C). For seedling growth, after 7 days, under control conditions, Si showed no significant effect on shoot length and root length of ZH11. Under drought stress, both shoot and root growth were significantly inhibited, shoot length decreased to about 0.38 cm, and root length to about 1.36 cm. However, silicon application markedly alleviated this inhibition, increasing shoot length to about 0.61 cm and root length to about 2.95 cm (Figure 1D–F). Similar trends were observed for the drought-tolerant variety Hanyou 73, with drought stress inhibiting germination and seedling growth, and Si application effectively relieving these inhibitory effects (Figure 2). Although Hanyou 73 possesses inherent drought tolerance, exogenous Si still significantly alleviated the inhibition of seed germination and early seedling growth under drought stress.

### 2.2. Silicon Promotes Seedling Establishment and Alleviates Growth Inhibition Under Water Deficit in Simulated Dry Direct Seeding

**Figure 3:**
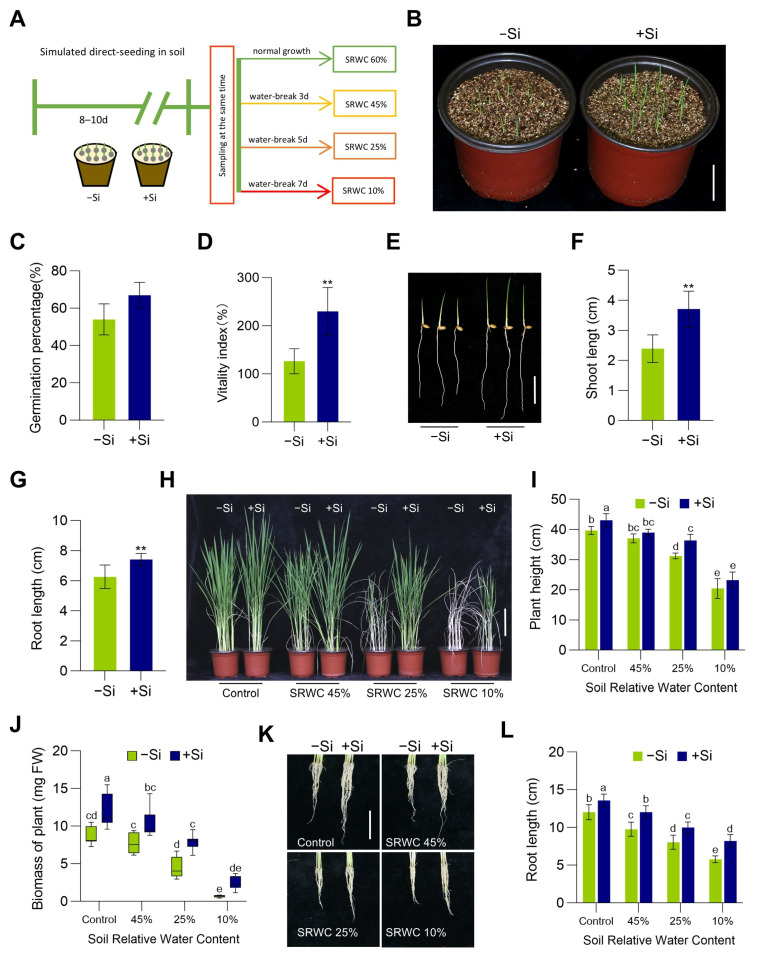
Si promotes rice seedling establishment and alleviates growth inhibition under drought stress in a simulated direct-seeding system. (**A**) Schematic diagram of the simulated direct-seeding experiment in soil. Rice seeds were sown in soil with (−Si) or without (+Si) silicon application. After germination, seedlings were subjected to different water-break treatments for 0, 3, 5, or 7 days to achieve soil relative water content (SRWC) of 60% (normal growth), 45%, 25%, and 10%, respectively. All samples were collected at the same time point. (**B**) Representative images of rice seedlings grown in soil with (+Si) or without (−Si) Si addition under SRWC 60% (control) conditions. Scale bar, 2 cm. (**C**,**D**) Germination percentage (**C**) and vitality index (**D**) of rice seeds under control conditions with or without Si. Data are presented as mean ± SD (n = 3). (**E**) Representative root images of 7-day-old rice seedlings under control conditions, with or without 2 mM Si addition. Scale bar, 1 cm. (**F**,**G**) Shoot length (**F**) and root length (**G**) of 7-day-old rice seedlings under control conditions with or without Si. (**H**) Representative images of rice plants grown under different SRWC conditions (Control, 45%, 25%, 10%) with (−Si) or without (+Si) Si addition. Scale bar, 10 cm. (**I**) Plant height of rice seedlings under different SRWC conditions. (**J**) Fresh biomass of rice plants under different SRWC conditions. (**K**) Representative root images of rice plants under different SRWC conditions with or without Si. Scale bar, 10 cm. (**L**) Root length of rice plants under different SRWC conditions. (**F**,**G**,**I**,**J**,**L**) Data are presented as mean ± SD (n = 15). Asterisks indicate significant differences between −Si and +Si groups under the same condition (** *p* < 0.01, Student’s *t*-test). Different lowercase letters indicate significant differences among groups (*p* < 0.05, one-way ANOVA followed by Tukey’s test).

**Figure 4:**
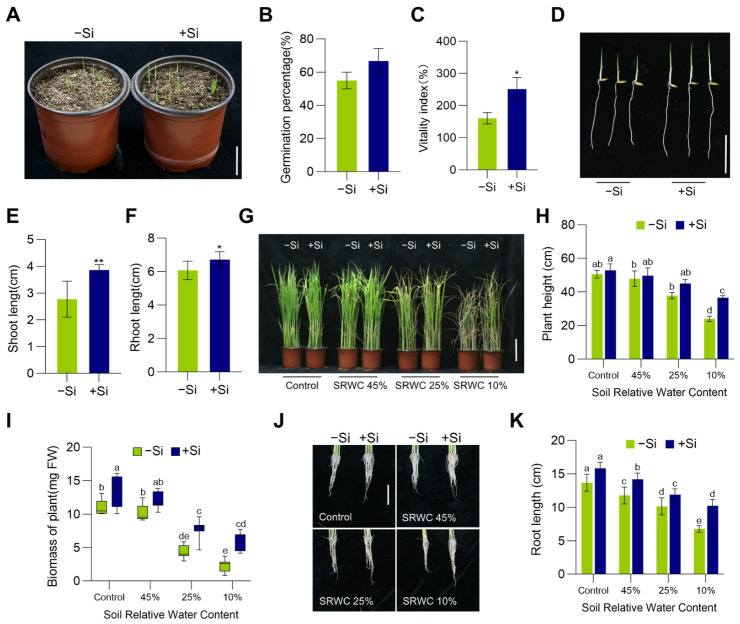
Si promotes rice seedling establishment and alleviates growth inhibition under drought stress in a simulated direct-seeding system. (**A**) Representative images of rice seedlings grown in soil with (+Si) or without (−Si) Si addition under SRWC 60% (control) conditions. Scale bar, 2 cm. (**B**,**C**) Germination percentage (**B**) and vitality index (**C**) of rice seeds under control conditions with or without Si. Data are presented as mean ± SD (n = 3). (**D**) Representative root images of 7-day-old rice seedlings under control conditions, with or without 2 mM Si addition. Scale bar, 1 cm. (**E**,**F**) Shoot length (**E**) and root length (**F**) of 7-day-old rice seedlings under control conditions with or without Si. (**G**) Representative images of rice plants grown under different SRWC conditions (Control, 45%, 25%, 10%) with (−Si) or without (+Si) Si addition. Scale bar, 10 cm. (**H**) Plant height of rice seedlings under different SRWC conditions. (**I**) Fresh biomass of rice plants under different SRWC conditions. (**J**) Representative root images of rice plants under different SRWC conditions with or without Si. Scale bar, 10 cm. (**K**) Root length of rice plants under different SRWC conditions. (**E**,**F**,**H**,**I**,**K**) Data are presented as mean ± SD (n = 15). Asterisks indicate significant differences between −Si and +Si groups under the same condition (* *p* < 0.05, ** *p* < 0.01, Student’s *t*-test). Different lowercase letters indicate significant differences among groups (*p* < 0.05, one-way ANOVA followed by Tukey’s test).

To further simulate actual DDS conditions, we compared seedling establishment and growth responses in the two rice varieties (ZH11 and Hanyou 73) under +Si and –Si conditions (Figure 3A). Under normal watering (60% SRWC), Si treatment significantly increased seed vigor index, shoot length, and root length in both varieties, but had no significant effect on germination percentage (Figure 3B–G and Figure 4A–F). With increasing drought severity, particularly under moderate (25%) and severe (10%) drought, plant height was significantly inhibited in both varieties; however, Si treatment effectively mitigated this suppression (Figure 3H,I and Figure 4G,H). At the same SRWC, Si-treated plants had significantly greater fresh weight and root length than untreated plants (Figure 3J–L and Figure 4I–K).

### 2.3. Silicon Enhances Photosynthetic Capacity Under Simulated Dry Direct Seeding in Rice

**Figure 5:**
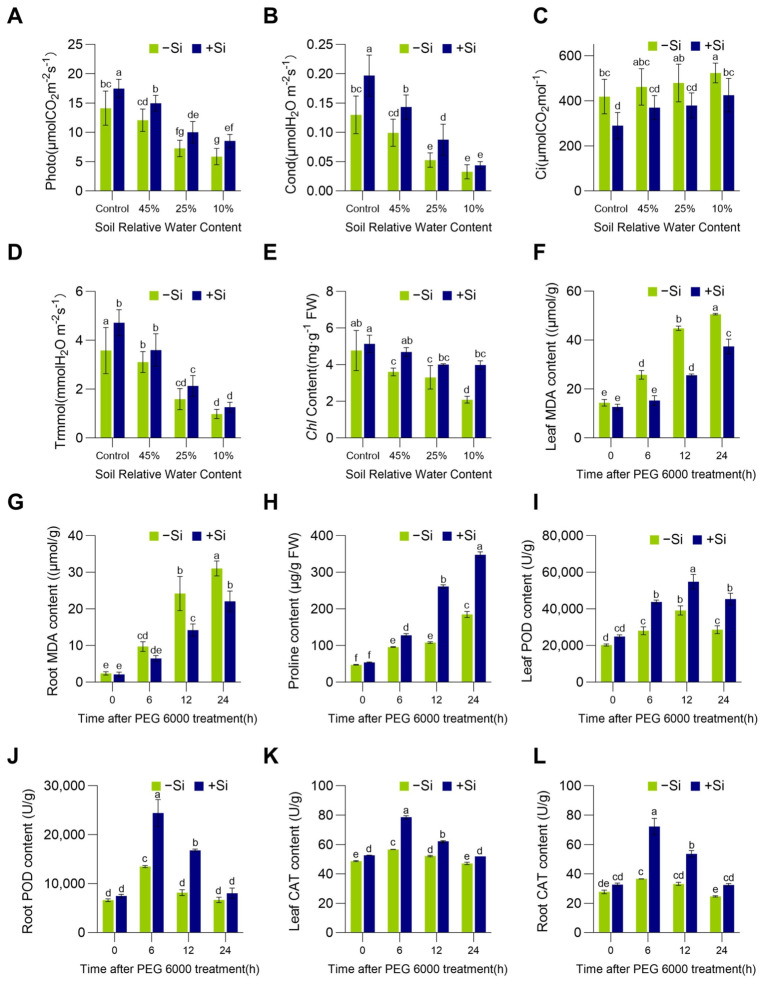
Effects of Si on photosynthetic parameters and physiological responses of rice under drought stress. (**A**–**D**) Photosynthetic parameters of rice plants grown under different soil relative water content (SRWC) conditions with or without Si application: (**A**) Net photosynthetic rate (Photo), (**B**) Stomatal conductance (Cond), (**C**) Intercellular CO_2_ concentration (Ci), (**D**) Transpiration rate (Trmmol). (**E**) Chlorophyll (Chl) content in rice leaves under different SRWC conditions with or without Si. (**F**–**L**) Physiological responses of rice seedlings under 20% PEG 6000-induced osmotic stress with or without Si application over time (0, 6, 12, 24 h): (**F**) Leaf malondialdehyde (MDA) content, (**G**) Root MDA content. (**H**) Proline content, (**I**) Leaf peroxidase (POD) activity, (**J**) Root POD activity, (**K**) Leaf catalase (CAT) activity, (**L**) Root CAT activity. (**A**–**D**) Data are presented as mean ± SD (n = 15). (**E**–**L**) Data are presented as mean ± SD (n = 3). Different lowercase letters indicate significant differences among groups (*p* < 0.05, one-way ANOVA followed by Tukey’s test).

To further explore the physiological mechanisms by which Si alleviates drought stress, we measured photosynthetic parameters (net photosynthetic rate, stomatal conductance, intercellular CO_2_ concentration, transpiration rate) of ZH11 plants under different SRWC conditions (60%, 45%, 25%, and 10%) in the simulated DDS system with or without Si application. Except for intercellular CO_2_ concentration, all measured parameters decreased with increasing drought severity (Figure 5A–D). At the same SRWC, Si-treated plants displayed significantly higher values for each parameter than untreated plants. Specifically, Si treatment effectively alleviated the drought-induced decrease in net photosynthetic rate (Figure 5A), increased stomatal conductance (Figure 5B), maintained higher intercellular CO_2_ concentration (Figure 5C), and promoted transpiration (Figure 5D). Si treatment also significantly slowed drought-induced chlorophyll degradation, allowing leaves to maintain higher chlorophyll content (Figure 5E).

### 2.4. Silicon Application Enhances Drought Tolerance by Activating the Antioxidant System and Osmotic Regulation in Rice

To reveal the physiological mechanisms by which Si alleviates water deficit in rice, we measured malondialdehyde (MDA) content, proline accumulation, and antioxidant enzyme activities in leaves and roots of rice plants with or without Si application under drought stress simulated by 20% PEG-6000. With increasing duration of simulated drought, MDA content increased markedly in the leaves and roots of Si-untreated plants, indicating progressive membrane lipid peroxidation. Si treatment significantly reduced MDA content in both leaves and roots, showing that Si effectively alleviates drought-induced oxidative damage (Figure 5F,G). Regarding osmotic regulation, drought stress induced proline accumulation, and Si treatment further promoted proline synthesis, resulting in higher proline content at the same time points (Figure 5H). For the antioxidant enzyme system, Si treatment maintained higher POD activity in leaves (Figure 5I) and roots (Figure 5J), as well as higher CAT activity in the leaves (Figure 5K) and roots (Figure 5L) at all time points examined. Quantitative real-time PCR analysis showed that Si treatment significantly upregulated the expression of several key drought-responsive genes (Supplementary Figure S1). Specifically, the expression levels of *SNAC1* and *DREB1A* were significantly higher in the +Si group relative to the –Si group, the expression of *SKIPa* was also significantly upregulated in the +Si group, and the upregulation of *P5CS2*, which encodes a key enzyme for proline synthesis, was consistent with the trend of proline accumulation.

### 2.5. The ABA Signaling Pathway Is Necessary for Silicon-Enhanced Drought Tolerance in Rice

ABA is a core hormone in the plant response to drought stress, playing critical regulatory roles by inducing stomatal closure to reduce transpiration water loss, regulating the expression of stress-responsive genes, and coordinating physiological adaptations [9]. To investigate whether the ABA pathway is involved in Si-mediated drought tolerance in rice, we first examined the expression changes in genes involved in ABA synthesis and signal transduction under simulated drought stress in rice plants with or without Si application (Supplementary Figure S2). Simulated drought treatment significantly upregulated the expression of ABA synthesis-related genes (*ABA1*, *ABA2*, *MHZ5*) and signaling-related genes (*ABI3*, *ABI5*, *bZIP23*). Moreover, the expression levels of ABA synthesis genes (*ABA1*, *ABA2*, *MHZ5*) and the ABA signaling gene *ABI3* were significantly higher in the +Si group than those in the corresponding –Si group, with the most pronounced upregulation of *ABI3* observed at 9 h of drought treatment, indicating that Si treatment upregulates the expression of genes involved in ABA synthesis and signaling.

**Figure 6:**
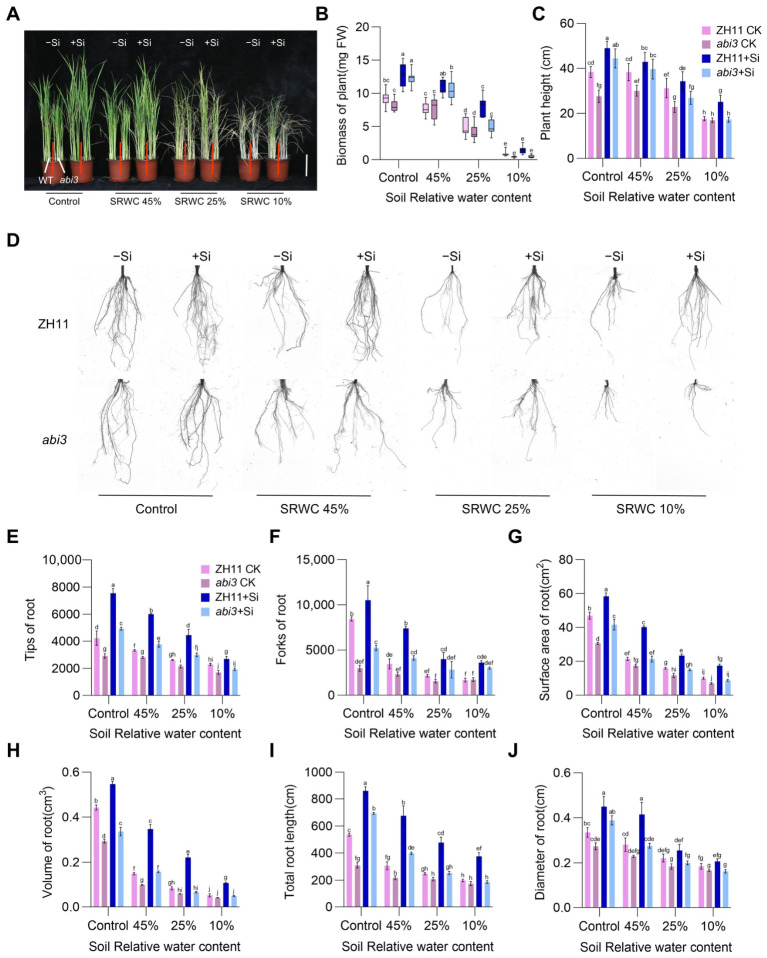
Si affects rice plant growth and root system architecture under different soil water conditions. (**A**) Phenotypes of ZH11 (wild-type) and *abi3* mutant rice plants after 7 days of treatment under different soil relative water content (SRWC: Control, 45%, 25%, 10%), with or without 2 mM Si addition. Scale bars, 10 cm. (**B**) Shoot fresh biomass of rice plants under different treatments. (**C**) Plant height of rice plants under different treatments. (**D**) Scanned images of rice root systems under different treatments. (**E**–**J**) Quantitative analysis of root system architecture parameters under different treatments: (**E**) root tips, (**F**) root forks, (**G**) root surface area, (**H**) root volume, (**I**) total root length, (**J**) root diameter. (**B**,**C**) Data are presented as mean ± SE (n = 15). (**E**–**J**) Data are presented as mean ± SE (n = 3). Different lowercase letters indicate significant differences among groups (*p* < 0.05, one-way ANOVA followed by Tukey’s test).

To further validate the necessity of the ABA signaling pathway in Si-mediated drought tolerance, we performed simulated DDS experiments using the ABA signaling mutant *abi3*, comparing the growth performance of ZH11 and *abi3* under +Si and –Si conditions. Phenotypic observation showed that under normal watering, both ZH11 and *abi3* plants grew well, and Si treatment significantly increased biomass and plant height in both genotypes (Figure 6A–C). Decreasing SRWC significantly inhibited the growth of both ZH11 and *abi3*, as reflected by the significant reductions in biomass and plant height relative to the control groups. At SRWC of 45%, 25%, and 10%, Si treatment significantly increased plant height and biomass in ZH11 compared with the –Si control, indicating that exogenous Si effectively ameliorated the reduction in biomass and plant height under water deficit. In contrast, Si did not alleviate the inhibition of biomass and plant height in the *abi3* mutant under water deficit (25% and 10% SRWC).

Further analysis of root architecture parameters (Figure 6E–J) showed that under normal watering, Si significantly increased all six root architecture parameters in both ZH11 and *abi3*. Under drought stress, Si treatment significantly increased the number of root tips and forks, root surface area, root volume, total root length, and root diameter in ZH11. In the *abi3* mutant, however, the enhancing effects of Si on these root parameters were markedly attenuated compared with ZH11. This indicates that the ABI3-mediated ABA signaling pathway is necessary for Si-promoted root development and Si-enhanced drought tolerance.

### 2.6. The JA Signaling Pathway Is Necessary for Silicon-Enhanced Drought Tolerance in Simulated Dry Direct Seeding Rice

JA is an important hormone in the plant response to drought stress. It enhances plant drought tolerance by activating downstream stress-responsive gene expression through transcription factors such as MYC2, promoting stomatal closure to reduce water loss, and regulating the reactive oxygen species scavenging system and osmotic balance [15,16]. To investigate whether the JA pathway is involved in Si-mediated drought tolerance in rice, we first examined the expression changes in genes involved in JA synthesis and signal transduction under simulated drought stress (Supplementary Figure S3). Si treatment significantly upregulated the expression of the JA synthesis genes *AOS2*, *AOS3*, *JAR1*, and *JAR2*, as well as the JA signaling genes *MYC2* and *COI1a*, indicating that Si treatment upregulates the expression of genes involved in JA synthesis and signaling.

**Figure 7:**
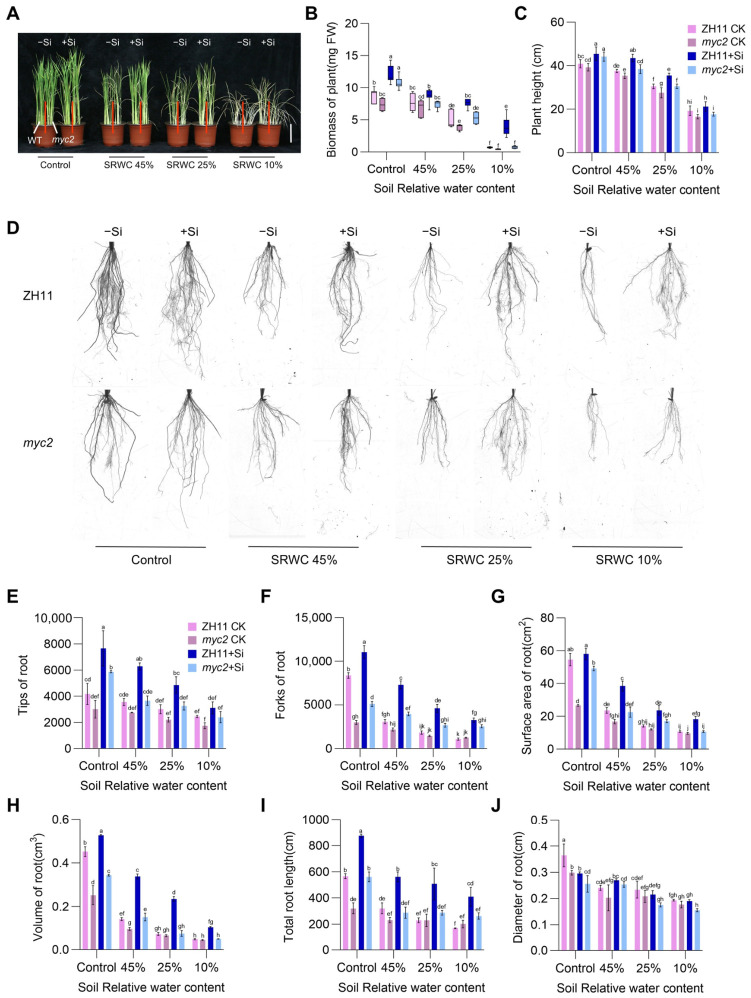
Si affects rice plant growth and root system architecture under different soil water conditions. (**A**) Phenotypes of ZH11 (wild-type) and *myc2* mutant rice plants after 7 days of treatment under different soil relative water content (SRWC: Control, 45%, 25%, 10%), with or without 2 mM Si addition. Scale bars, 10 cm. (**B**) Shoot fresh biomass of rice plants under different treatments. (**C**) Plant height of rice plants under different treatments. (**D**) Scanned images of rice root systems under different treatments. (**E**–**J**) Quantitative analysis of root system architecture parameters under different treatments: (**E**) root tips, (**F**) root forks, (**G**) root surface area, (**H**) root volume, (**I**) total root length, (**J**) root diameter. (**B**,**C**) Data are presented as mean ± SE (n = 15). (**E**–**J**) Data are presented as mean ± SE (n = 3). Different lowercase letters indicate significant differences among groups (*p* < 0.05, one-way ANOVA followed by Tukey’s test).

To further validate the necessity of the JA signaling pathway in Si-mediated drought tolerance, we performed simulated DDS experiments using the JA signaling mutant *myc2*. We set different SRWC conditions (control, 45%, 25%, 10%) and compared the growth performance of ZH11 and *myc2* under +Si and –Si conditions. Under normal watering, both ZH11 and *myc2* plants grew well, and Si treatment significantly increased biomass and plant height in both genotypes. With increasing drought severity, Si treatment significantly increased plant height and biomass in ZH11, whereas the *myc2* mutant exhibited a significantly weaker response to Si treatment (Figure 7A–D).

Analysis of root architecture (Figure 7E–J) showed that under water deficit, Si application improved root growth with significant increases in the number of root tips, number of forks, root surface area, root volume, total root length, and root diameter in ZH11. In the *myc2* mutant, however, such Si-mediated improving effects on these root parameters were markedly attenuated, and for some parameters, no significant differences were observed. This indicates that the MYC2-mediated JA signaling pathway is necessary for Si-promoted root development and Si-enhanced drought tolerance.

## 3. Discussion

The central finding of this study is that exogenous Si significantly enhances the adaptation of rice to drought stress under simulated DDS conditions through a multi-mechanistic synergy that integrates osmotic regulation, activation of antioxidant defenses, maintenance of photosynthetic function, and ABA and JA signal transduction.

Seed germination and early seedling establishment are critical periods determining the quality of seedling establishment in DDS rice, and they are the developmental stages most sensitive to drought stress [1]. Our study showed that under simulated drought with 20% PEG-6000, exogenous Si treatment increased the germination percentage of ZH11 seeds from 73.3% to 100% and significantly promoted radicle elongation. This result is consistent with the study by Abd-El-Aty et al. [16] in rice, which reported that exogenous Si application significantly increased germination percentage and seedling growth parameters under drought stress across multiple rice genotypes. The promoting effect of Si in our study was particularly pronounced for root length, which is consistent with the known role of Si in promoting root cell division and elongation [16]. In the simulated DDS experiments, as SRWC decreased from 45% to 10%, seedling height and biomass accumulation in rice were significantly inhibited, and Si application significantly countered this decrease. This finding is consistent with the study by Jiang et al. in dry-cultivated rice, which showed that Si treatment enhanced the adaptation of rice to drought by promoting flavonoid biosynthesis and the accumulation of osmotic regulatory substances [9]. The significant alleviating effects of Si treatment in both rice varieties indicate that Si-induced drought tolerance is broadly applicable across varieties.

One of the most direct damaging effects of drought stress on plants is the inhibition of photosynthesis [17]. In this study, as SRWC decreased, the net photosynthetic rate, stomatal conductance, and chlorophyll content of rice seedlings all decreased significantly, consistent with previous findings in rice [18]. Si treatment significantly alleviated the decreases in these photosynthetic parameters. The protective effect of Si on photosynthesis may involve two mechanisms. On the one hand, the deposition of Si in leaf epidermal cells forms a silicified layer that effectively maintains leaf erectness, reducing the decrease in light capture efficiency caused by wilting [19]. On the other hand, Si protects the structural integrity of chloroplasts and inhibits drought-induced chlorophyll degradation [20]. The higher transpiration rate observed in Si-treated plants in our study may be attributed to improved root water uptake capacity, which maintains better leaf water status [21].

Excessive reactive oxygen species (ROS) accumulation under drought stress causes cellular damage [22]. In our study, prolonged simulated drought progressively increased MDA content in rice leaves and roots, indicating membrane lipid peroxidation. Si treatment significantly reduced MDA accumulation while increasing POD and CAT activities. Consistent with Du et al. [23], who reported that Si upregulates the catalase gene *OsCatB*, enhances ROS scavenging, and maintains ROS homeostasis. Temporally, POD and CAT activities in Si-treated plants peaked at 6–12 h of drought and remained significantly higher than controls, indicating that Si rapidly boosts antioxidant defense [7]. Si treatment promoted proline accumulation, correlating with upregulated expression of proline synthesis gene *P5CS2*, which provides a molecular explanation. Similar findings were reported by Abd-El-Aty et al. [16] with exogenous proline application.

Phytohormones play central regulatory roles in the drought stress response [24]. Our study showed that Si treatment significantly upregulated the expression of ABA synthesis genes (*ABA1*, *ABA2*, *MHZ5*) and the signaling gene (*ABI3*). Controlled-watering pot experiments using the ABA signaling mutant *abi3* further confirmed that in the absence of *ABI3*, the promoting effects of Si on plant height, biomass, and root morphological parameters were significantly attenuated. This indicates that the ABA signaling pathway is necessary for the drought tolerance function of Si. This conclusion is highly consistent with a recent study by Du et al. [23], which clearly demonstrated that the ABA signaling pathway is necessary for Si-mediated drought tolerance, and that Si treatment upregulates the expression of the ABA biosynthesis gene *NCED3* as well as the ABA-responsive genes *DREB2A* and *LEA5*. In our study, Si treatment also significantly upregulated the expression of JA synthesis genes *AOS2*, *AOS3*, *JAR1*, *JAR2* and signaling pathway genes (*MYC2* and *COI1a*), and the JA signaling mutant *myc2* exhibited a significantly weaker response to Si treatment than ZH11. Our finding that the promoting effects of Si on root parameters—including number of root tips, number of forks, root surface area, root volume, and total root length—were significantly attenuated in both the *abi3* and *myc2* mutants suggests that the ABA and JA signaling pathways may jointly participate in Si-mediated regulation of root development [25,26]. This finding has important theoretical implications, as root development in DDS rice directly affects the capacity for water uptake from deeper soil layers [24]. The study by Jiang et al. [4] also confirmed that Si treatment promotes flavonoid accumulation and synthesis of osmotic regulatory substances in the roots of dry-cultivated rice, thereby enhancing root adaptation to drought.

Although this study has systematically revealed the physiological and molecular mechanisms by which Si enhances drought tolerance in DDS rice. Several limitations of this study should be acknowledged. First, subsequent field experiments are needed to validate the actual yield-enhancing effects of Si fertilizer [5]. Second, our analyses focused mainly on the seed germination and seedling stages. The effects of Si on drought tolerance and their relationship with yield traits throughout the entire rice growth cycle require further investigation. Third, while ABI3 and MYC2 are master transcription factors at central nodes of their respective signaling pathways, the use of only one mutant per pathway limits the strength of genetic validation. Future studies using additional mutants (e.g., *abi5*, *nced3* for ABA; *coi1*, *jar1* for JA) would provide complementary evidence. Furthermore, while our loss-of-function analyses confirm that both ABA and JA signaling pathways are individually necessary for Si-enhanced drought tolerance, whether these pathways function independently or synergistically remains to be determined. Future studies using the *abi3/myc2* double mutant will help elucidate potential additive or epistatic interactions between ABA and JA signaling in Si-mediated root development and drought adaptation. Nevertheless, this study provides a theoretical basis for the application of Si fertilizer in DDS rice cultivation and offers new strategies for the genetic improvement of crop drought tolerance through hormone signaling pathways.

## 4. Materials and Methods

### 4.1. Plant Materials and Growth Conditions

The rice materials used in this study included the conventional japonica variety Zhonghua 11 (ZH11), the nationally approved water-saving and drought-tolerant hybrid variety Hanyou 73, the ABA signaling mutant *abi3*, and the JA signaling mutant *myc2*. The Si-free rice nutrient solution was prepared according to the International Rice Research Institute (IRRI) formula, and the +Si treatment consisted of the same nutrient solution supplemented with 2 mM sodium silicate (Na_2_SiO_3_, Macklin Biochemical Technology Co., Ltd., Shanghai, China). The pH of all nutrient solutions was adjusted to 5.8 using HCl or NaOH before use.

### 4.2. Seed Germination Experiment Under Simulated Drought

Plump, uniformly sized rice seeds were surface-sterilized with 2% sodium hypochlorite for 15 min and then subjected to the following four treatments: (1) pure water (CK); (2) 2 mM Na_2_SiO_3_ solution; (3) 20% PEG-6000; (4) 20% PEG-6000 + 2 mM Na_2_SiO_3_ solution. Three biological replicates were used per treatment, with 30 seeds per replicate. PEG-6000 was purchased from Macklin Biochemical Technology Co., Ltd. (Shanghai, China). Seeds were germinated in a growth chamber with a 12 h light (30 °C)/12 h dark (25 °C) photoperiod, and the solutions were replaced every 24 h. Germination percentage and germination index were recorded daily for 5 days, and root length and shoot length were measured after 7 days. Germination percentage and germination index were calculated according to standard methods. Germination percentage was determined as the proportion of seeds that had germinated by the end of the 5-day experimental period, expressed as a percentage of total seeds tested. Germination index was calculated using the formula: Germination index = Σ (Gt/Dt), where Gt is the number of newly germinated seeds on day t, and Dt is the corresponding day of germination (1–5).

### 4.3. Pot Experiment with Different Soil Relative Water Contents Simulating DDS

Potting soil consisted of a 1:1 (*v*/*v*) mixture of nutrient soil and vermiculite, which was air-dried and then placed into pots. The mixed soil was saturated with either Si-free nutrient solution or 2 mM Si-containing nutrient solution. Thirty sterilized seeds were sown per pot and covered with a layer of vermiculite. At the three-leaf stage, water treatments were initiated by setting soil relative water contents (SRWC) to 60% (control), 45%, 25%, and 10%, with three biological replicates per treatment and 30 plants per replicate. The corresponding nutrient solution was added daily to maintain the designated SRWC, which was monitored using a soil moisture meter. The experiment was terminated when seedlings in the 10% SRWC group died, after which plant height, root length, and shoot fresh weight were measured.

### 4.4. Photosynthetic Parameter and Chlorophyll Content Measurements

Photosynthetic parameters were measured between 9:00 and 12:00 a.m. using a LI-6400XT portable photosynthesis system on the youngest fully expanded leaves. The measurement conditions were: light intensity 600 μmol·m^−2^·s^−1^, flow rate 500 μmol·s^−1^, leaf temperature 28 °C, and chamber CO_2_ concentration 320 μmol·mol^−1^. Parameters measured included net photosynthetic rate, stomatal conductance, intercellular CO_2_ concentration, and transpiration rate, with 15 biological replicates per treatment. For chlorophyll content, leaf samples were ground and homogenized in 80% acetone, centrifuged, and the absorbance of the supernatant was measured at 645 nm and 663 nm to calculate chlorophyll a, chlorophyll b, and total chlorophyll content, with three biological replicates per treatment.

### 4.5. Proline Content Determination

Proline content was measured using the acid ninhydrin colorimetric method. Samples were extracted with 3% sulfosalicylic acid, then reacted with glacial acetic acid and acid ninhydrin reagent in a boiling water bath for 30 min. After cooling, toluene was added for extraction, and the absorbance of the toluene phase was measured at 520 nm. Proline content was calculated using a standard curve, with three biological replicates per treatment.

### 4.6. Malondialdehyde Content Determination

Samples were extracted with 10% trichloroacetic acid, then reacted with 0.6% thiobarbituric acid in a boiling water bath for 15 min. After rapid cooling and centrifugation, the absorbance of the supernatant was measured at 532 nm, 600 nm, and 450 nm to calculate MDA content, with three biological replicates per treatment.

### 4.7. Antioxidant Enzyme Activity Assays

POD activity was measured using the guaiacol method. The reaction mixture contained 50 mmol·L^−1^ phosphate buffer (pH 7.0), 20 mmol·L^−1^ guaiacol, and 40 mmol·L^−1^ H_2_O_2_. The reaction was initiated by adding 0.1 mL of enzyme extract, and the increase in absorbance at 470 nm was monitored continuously for 3 min (every 30 s) at 25 °C. One unit of POD activity (U) was defined as an increase of 0.01 in absorbance per minute, and activity was expressed as U·mg^−1^ protein.

CAT activity was measured using the ultraviolet absorption method. The reaction mixture contained 50 mmol·L^−1^ phosphate buffer (pH 7.0) and 0.1 mL of enzyme extract. The reaction was initiated by adding 20 mmol·L^−1^ H_2_O_2_, and the decrease in absorbance at 240 nm was monitored continuously for 3 min (every 30 s) at 25 °C. One unit of CAT activity (U) was defined as a decrease of 0.01 in absorbance per minute, and activity was expressed as U·mg^−1^ protein. Three biological replicates were used per treatment for both assays.

### 4.8. RNA Extraction and Quantitative Real-Time PCR

Rice seedlings were grown in Si-free or 2 mM Si-containing nutrient solution until the five-leaf stage and then subjected to simulated drought stress by adding 20% PEG-6000 in the corresponding nutrient solution. Shoot and root samples were collected at 0, 3, 6, 9, 12, and 24 h after treatment, immediately frozen in liquid nitrogen, and stored at −80 °C for subsequent gene expression analysis and physiological measurements, with three biological replicates per time point. Total RNA was extracted from rice leaves using the Trizol method and reverse-transcribed into cDNA. Quantitative real-time PCR was performed using SYBR Green dye. The rice housekeeping gene was used as an internal control, and relative expression levels were calculated using the 2^−ΔΔCt^ method. RNA extraction and qRT-PCR reagents were purchased from LABLEAD Biotechnology Co., Ltd. (Beijing, China). Primer sequences are provided in Supplementary Table S1.

### 4.9. Root Architecture Analysis

Washed rice roots were scanned using an Epson Expression LA2400 scanner to obtain grayscale images. Root architecture parameters, including total root length, root surface area, root diameter, root volume, number of root tips, and number of forks, were analyzed using the WinRHIZO root analysis system, with three biological replicates per treatment.

### 4.10. Statistical Analysis

All experimental data were first organized using Excel and then statistically analyzed using IBM SPSS Statistics 27. Comparisons between two groups were performed using independent-samples *t*-tests, with significance levels set at * *p* < 0.05 (significant) and ** *p* < 0.01 (highly significant). Multiple comparisons were performed using one-way ANOVA followed by Tukey’s post hoc test. Graphs were generated using GraphPad Prism 10.

## 5. Conclusions and Future Perspectives

In conclusion, this study demonstrates that exogenous silicon alleviates drought stress and enhances seedling establishment in simulated dry direct-seeding rice through a multi-mechanistic synergy that integrates osmotic regulation, activation of antioxidant defenses, maintenance of photosynthetic function, and activation of both ABA and JA signaling pathways. Genetic evidence using the *abi3* and *myc2* mutants confirms that both ABA and JA signaling pathways are individually necessary for Si-mediated drought tolerance.

Future research should focus on field validation of silicon fertilizer effects under actual dry direct seeding conditions across multiple growing seasons and different soil types. Future research should also include drought recovery assessments after rewatering to evaluate the lasting effects of Si pre-treatment on post-drought recovery capacity. Further genetic analysis, including the generation and characterization of the *abi3/myc2* double mutant, is needed to elucidate potential additive or synergistic interactions between ABA and JA signaling pathways. Direct quantification of ABA and JA hormone levels will also be important to determine whether silicon modulates hormone accumulation or signaling competence. Additionally, the long-term effects of silicon application on drought tolerance and yield traits throughout the entire rice growth cycle warrant investigation. Collectively, these efforts will provide a more comprehensive understanding of silicon’s mode of action and facilitate its practical application in dry direct seeding rice production systems.

## Data Availability

The original contributions presented in this study are included in the article/Supplementary Materials. Further inquiries can be directed to the corresponding authors.

## Supplementary Materials

The following supporting information can be downloaded at https://www.mdpi.com/article/10.3390/plants15121813/s1. Supplemental Figure S1: Si modulates the expression of drought-responsive genes in rice under simulated drought stress. Time-course analysis of the relative expression of drought-responsive genes in rice seedlings treated with 20% PEG 6000 for 0, 3, 6, 9, 12, and 24 h, with or without 2 mM Si addition. The genes analyzed include: *SNAC1* (A), *DREB1A* (B), *SKIPa* (C), and *P5C52* (D). The relative expression levels were normalized to the reference gene Actin and presented as fold change relative to the 0 h −Si control. Data are presented as mean ± SD (n = 3). Asterisks indicate significant differences between −Si and +Si groups at the same time point (* *p* < 0.05, ** *p* < 0.01, Student’s *t*-test). Supplemental Figure S2: Si modulates the expression of ABA biosynthesis and signaling-related genes in rice under simulated drought stress. Time-course analysis of the relative expression of ABA pathway genes in rice seedlings treated with 20% PEG 6000 for 0, 3, 6, 9, 12, and 24 h, with or without 2 mM Si addition. The genes include: *ABA1* (A), *ABA2* (B), *MHZ5* (C), *ABI3* (D), *ABI5* (E), and *bZIP23* (F). The relative expression levels were normalized to the reference gene Actin and presented as fold change relative to the 0 h −Si control. Data are presented as mean ± SD (n = 3). Different lowercase letters indicate significant differences among groups (*p* < 0.05, one-way ANOVA followed by Tukey’s test). Supplemental Figure S3: Si modulates the expression of JA biosynthesis and signaling-related genes in rice under simulated drought stress. Time-course analysis of the relative expression of JA pathway genes in rice seedlings treated with 20% PEG 6000 for 0, 3, 6, 9, 12, and 24 h, with or without 2 mM Si addition. The genes include: the JA biosynthetic genes *AOS2* (A), *AOS3* (B), *JAR1* (C), *JAR2* (D), and the JA signaling-related genes *MYC2* (E) and *COI1a* (F). The relative expression levels were normalized to the reference gene Actin and presented as fold change relative to the 0 h −Si control. Data are presented as mean ± SD (n = 3). Different lowercase letters indicate significant differences among groups (*p* < 0.05, one-way ANOVA followed by Tukey’s test). Supplemental Table S1: List of primers used in this study.
