# Supplementary material for: Silicon Alleviates Drought Stress and Enhances Rice Seedling Establishment Under Simulated Dry Direct Seeding via Regulation of ABA and JA Signaling
Source: Plants (Basel). 2026 Jun 12;15(12):1813. doi: 10.3390/plants15121813 (PMC13306590; doi:10.3390/plants15121813)
Supplement: Supplementary file 1 [file plants-15-01813-s001.zip › plants-4356379-supplementary.pdf]

Supplemental Materials

Supplemental Figure S1. Si modulates the expression of drought-responsive genes in rice under simulated drought stress.

Time-course analysis of the relative expression of drought-responsive genes in rice seedlings treated with 20% PEG 6000 for 0, 3, 6, 9, 12, and 24 h, with or without 2 mM Si addition. The genes analyzed include: *SNAC1* (A), *DREB1A* (B), *SKIPa* (C), and *P5C52* (D). The relative expression levels were normalized to the reference gene Actin and presented as fold change relative to the 0 h -Si control. Data are presented as mean ± SD (n = 3). Asterisks indicate significant differences between -Si and +Si groups at the same time point (**p* < 0.05, ***p* < 0.01, Student's *t*-test).

Supplemental Figure S2. Si modulates the expression of ABA biosynthesis and signaling-related genes in rice under simulated drought stress.

Time-course analysis of the relative expression of ABA pathway genes in rice seedlings treated with 20% PEG 6000 for 0, 3, 6, 9, 12, and 24 h, with or without 2 mM Si addition. The genes include: *ABA1* (A), *ABA2* (B), *MHZ5* (C), *ABI3* (D), *ABI5* (E), and *bZIP23* (F). The relative expression levels were normalized to the reference gene Actin and presented as fold change relative to the 0 h -Si control. Data are presented as mean ± SD (n = 3). Different lowercase letters indicate significant differences among groups (*p* < 0.05, one-way ANOVA followed by Tukey's test).

Supplemental Figure S3. Si modulates the expression of JA biosynthesis and

signaling-related genes in rice under simulated drought stress.

Time-course analysis of the relative expression of JA pathway genes in rice seedlings treated with 20% PEG 6000 for 0, 3, 6, 9, 12, and 24 h, with or without 2 mM Si addition. The genes include: the JA biosynthetic genes *AOS2*(A), *AOS3*(B), *JAR1*(C), *JAR2*(D), and the JA signaling-related genes *MYC2*(E), and *COI1a*(F). The relative expression levels were normalized to the reference gene Actin and presented as fold change relative to the 0 h -Si control. Data are presented as mean ± SD (n = 3). Different lowercase letters indicate significant differences among groups ($p < 0.05$, one-way ANOVA followed by Tukey's test).

Supplemental Table S1. List of primers used in this study.

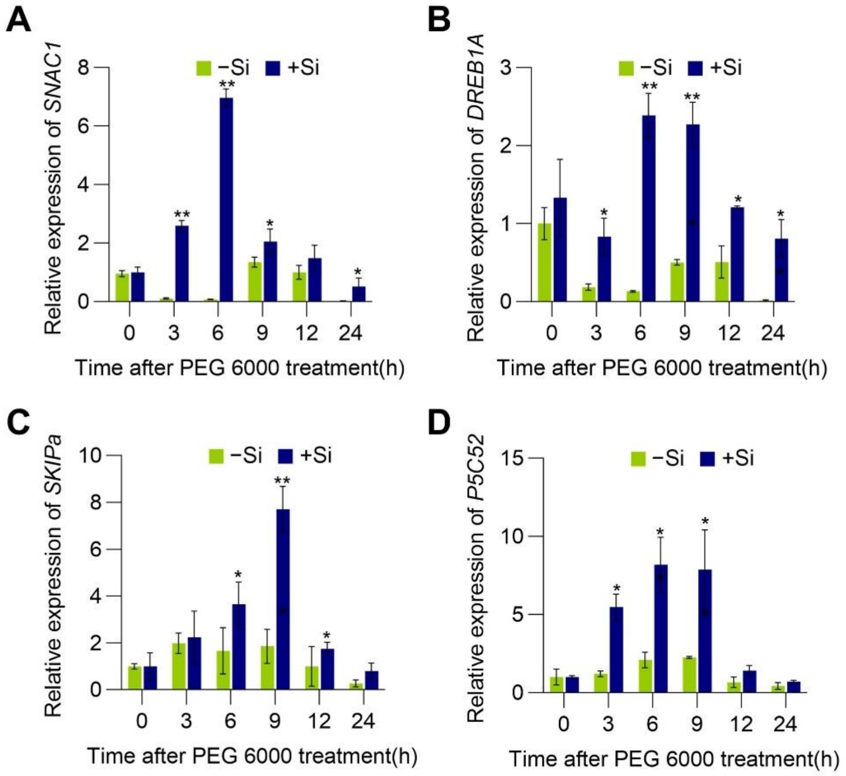

Supplemental Figure S1. Si modulates the expression of drought-responsive genes in rice under simulated drought stress.

Time-course analysis of the relative expression of drought-responsive genes in rice seedlings treated with 20% PEG 6000 for 0, 3, 6, 9, 12, and 24 h, with or without 2 mM Si addition. The genes analyzed include: *SNAC1* (A), *DREB1A* (B), *SKIPa* (C), and *P5C52* (D). The relative expression levels were normalized to the reference gene Actin and presented as fold change relative to the 0 h -Si control. Data are presented as mean ± SD (n = 3). Asterisks indicate significant differences between -Si and +Si groups at the same time point (**p* < 0.05, ***p* < 0.01, Student's *t*-test).

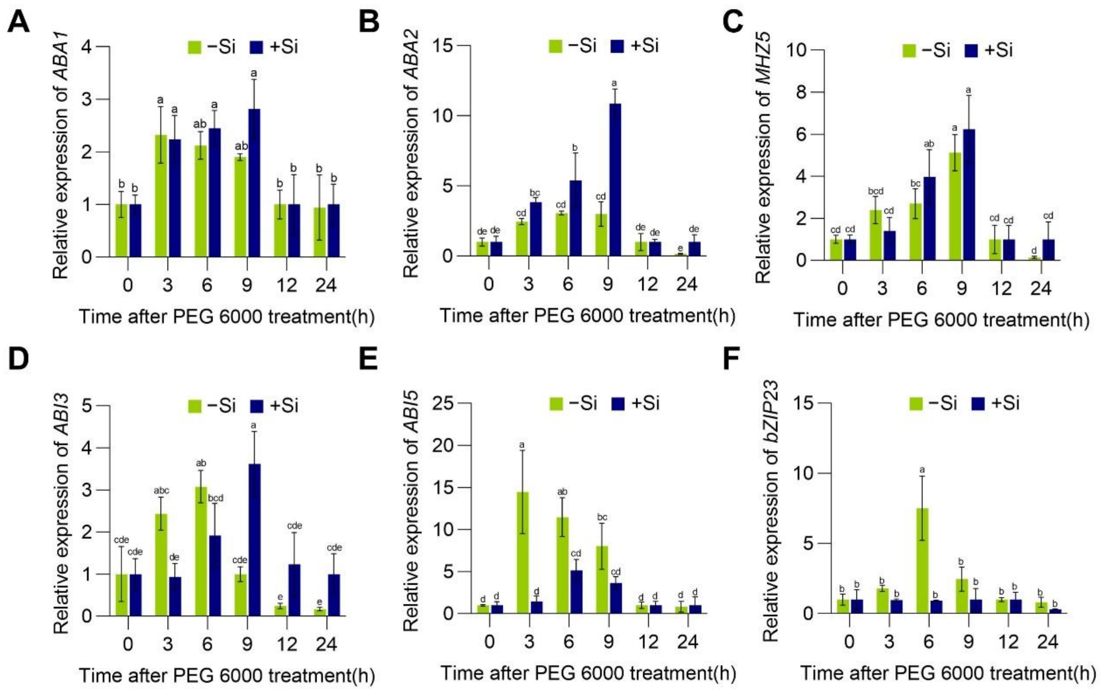

Supplemental Figure S2. Si modulates the expression of ABA biosynthesis and signaling-related genes in rice under simulated drought stress.

Time-course analysis of the relative expression of ABA pathway genes in rice seedlings treated with 20% PEG 6000 for 0, 3, 6, 9, 12, and 24 h, with or without 2 mM Si addition. The genes include: *ABA1* (A), *ABA2* (B), *MHZ5* (C), *ABI3* (D), *ABI5* (E), and *bZIP23* (F). The relative expression levels were normalized to the reference gene Actin and presented as fold change relative to the 0 h -Si control. Data are presented as mean ± SD (n = 3). Different lowercase letters indicate significant differences among groups ($p < 0.05$, one-way ANOVA followed by Tukey's test).

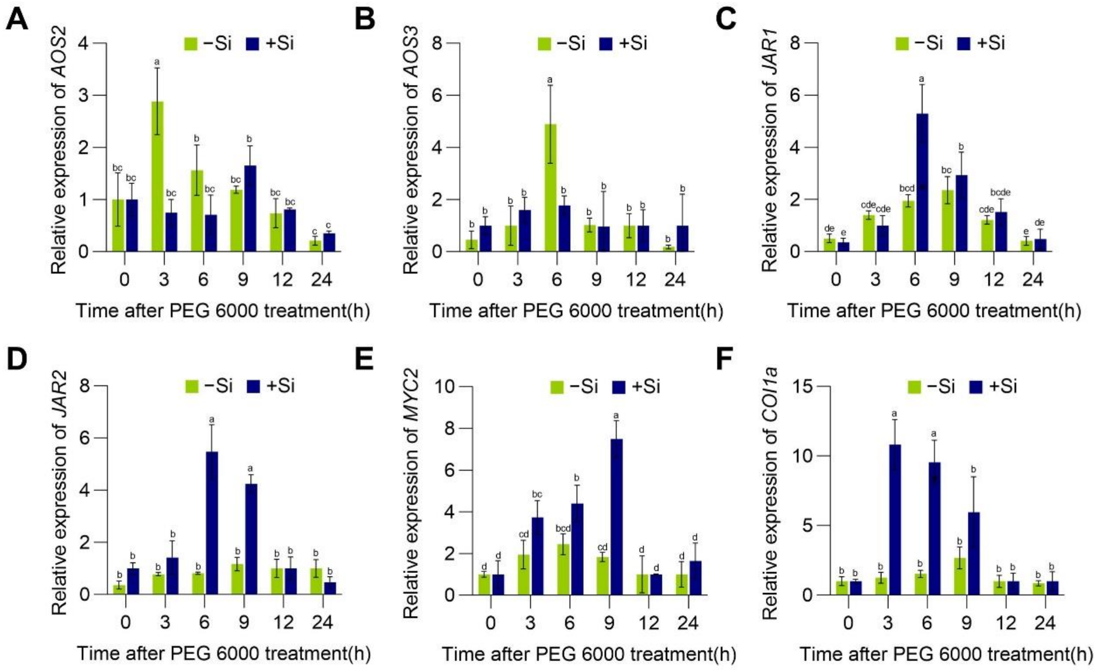

Supplemental Figure S3. Si modulates the expression of JA biosynthesis and signaling-related genes in rice under simulated drought stress.

Time-course analysis of the relative expression of JA pathway genes in rice seedlings treated with 20% PEG 6000 for 0, 3, 6, 9, 12, and 24 h, with or without 2 mM Si addition. The genes include: the JA biosynthetic genes *AOS2*(A), *AOS3*(B), *JAR1*(C), *JAR2*(D), and the JA signaling-related genes *MYC2*(E), and *COI1a*(F). The relative expression levels were normalized to the reference gene Actin and presented as fold change relative to the 0 h -Si control. Data are presented as mean ± SD (n = 3). Different lowercase letters indicate significant differences among groups ($p < 0.05$, one-way ANOVA followed by Tukey's test).

Table S1. List of primers for RT-qPCR used in this study.

Gene Name	Primer Sequence (5'-3')
Actin-F	ATCCTTCGTCTCGACCTTGC
Actin-R	TGGCAGTCTCCATTTCCTGG
SNAC1-F	GGTGGTGGTAGAGGTGCTGTT
SNAC1-R	GCGAAGAGCGACGAGTAGAAGT
DREB1A-F	GAGGCCGTCGAGGACTTCTT
DREB1A-R	TCCCAGCCCATGTCACTCAG
SKIPa-F	GGTGGTGGTAGAGGTGCTGTT
SKIPa-R	CGAATCCTGTCACGCTCAATCC
P5CS2-F	TTGGTCTAGGTGCTGAGGTTGG
P5CS2-R	CCTTGTCGCCGTTCACTACTTG
ABA1-F	GGTTGGTGCTGATGGAAT
ABA1-R	AAGTAGGTGGTCGGTCATA
ABA2-F	TTCGGTGGAAGAGGATGT
ABA2-R	GGTTGCTGTGTATGAATGAG
MHZ5-F	TGGTGGTGTTGGTGGTAT
MHZ5-R	AATGGTGGCAATCAGTATCA
ABI3-F	GATACGAAGGCGGTGATG
ABI3-R	TTCTGGAGGTGGTGGTTC
ABI5-F	ACACTAGGTGAGATGACACTTG
ABI5-R	CGTTTACCGGTCCTACTGGG
bZIP23-F	TTGTCGAGGAGAGACAGCGG
bZIP23-R	TGCTCCAACATTTCATCCTGC
AOS2-F	GGAGATGCTGTTCGGCTACCA
AOS2-R	GATTGACGGCGGAGGTTGAAG
AOS3-F	TCCTGCCACACATCGTCAAGT
AOS3-R	TGGTAGCCGAACAGCATCTCC
JAR1-F	CACGCTTCCACAACTCCACAC
JAR1-R	TCACTCGCATCGCCACTCAG
JAR2-F	GAGAAGAAGGCGACGAGGAGTA
JAR2-R	CGGCGATGCGGTCAATGTAG
MYC2-F	CCTCACCACCACCACCAACA
MYC2-R	TCGTCGCAGCCCTTGTAGTAG
COI1a-F	CAGAGCCAGCCAGCAACAAC
COI1a-R	TAGCAGAAGGCGACGGTGAC
